# The Molecular Basis for the Environmental Promotion of Neurodegenerative Disease

**DOI:** 10.3390/ijms24076209

**Published:** 2023-03-25

**Authors:** Stephen Bondy

**Affiliations:** Department of Environmental and Occupational Health, University of California, Irvine, CA 92617, USA; scbondy@uci.edu

Most neurodegenerative diseases have a relatively minor genetic component. Huntington’s disease is the only major exception, with the genetic penetrance being 100%. This implies that unknown environmental factors play a major role in determining the prevalence of defined neurological diseases in a specific population. Such agents consist of materials present in the general environment, such as those in air, water, or surrounding terrains.

This Special Issue is focused on the possible molecular mechanisms by which these exogenous influences can initiate or accelerate processes associated with brain aging. Such an expedition of normal events can lead to the premature emergence of distinctive disorders associated with brain aging. Mechanistic clues may be initiated by findings from clinical or epidemiological studies and followed up using more detailed analyses of animal and isolated cell models. Each of the chapters will elaborate on some aspects of the more common neurodegenerative disorders to better understand the molecular disruption that is involved.

This Special Issue is thus devoted to identifying pathways by which xenobiotic agents can play a role in the onset and progression of neurodegenerative disease. As a consequence of human industrial activity, there is increasing exposure to a wide range of materials in the environment. While these agents may be at low levels in comparison to work-related toxic exposures, they can be continuously present. Their persistent presence implies that they can uninterruptedly influence organismic metabolism, potentially over many years. This allows interaction with normal aging processes. By this means, aging can be accelerated. Many neurodegenerative disorders are based on an aging platform and are only expressed at a time when adulthood merges into gradual senescence. For the five contributions to this Special Issue, each use a selected toxicant to delineate those features of brain biology that serve as vulnerable loci for the induction of neurochemical lesions. The potential number of agents harmful to the functioning of the nervous system is very large. However, there are a smaller number of key foci that are especially susceptible to disruption by xenobiotics. It is important to identify those areas whose disruption can result in a significant decline in brain function. Since these are likely to include systems that are also adversely affected by normal aging, there is a likelihood of adverse synergistic interactions. The facilitation of normal age-related changes can lead to the premature emergence of specific neurodegenerative diseases.

The research report by Kim et al. [1] deals with the toxicity of isothiazolinones, a widely used class of anti-microbial agents present in many household and industrial agents. Using an isolated system of brain endothelial cells, mitochondrial malfunction and excess caspase-3-related apoptosis were noted. The impairment of these cells’ barrier function by 2-n-octyl-4-isothiazolin-3-one was inferred to reflect the potential for such agents damage the blood-brain barrier. The finding that many of the deleterious events effected by these chemicals are reversed by n-acetyl cysteine suggests that the broad range of sequelae following exposure to them may be consequent to oxidative stress.

The review by Huang et al. [2] addresses the neurotoxic agents that appear to promote the prevalence of Parkinson’s disease. In addition to pesticides and several heavy metals, traumatic brain injury increases the risk of incurring this disorder. A detailed examination of a range of agents known to be harmful to dopaminergic systems is presented. In general, elevated neuroinflammation and oxidative stress characterize parkinsonism, and these can be affected by the failure of effective mitochondrial functioning. Toxicants directed toward manganese can also lead to the misfolding of proteins, leading to the deposition of aggregates such as α-synuclein. The reasons for the particular susceptibility of the nigrostriatal pathway to a diverse range of chemicals remain unresolved but may involve the proneness of dopamine to oxidative damage.

The review by Thakur et al. [3] is focused on the neurotoxicity of inorganic arsenic. There is a detailed examination of its metabolism in the body and several key organic derivatives. Many detrimental systemic changes are brought about by chronic exposure to low levels of these salts. However, there may also be damage to the developing nervous system that is only manifested later in life. What may initially manifest as neurobehavioral deficits can later lead to neurodegenerative disorders such as Parkinson’s disease. Such progression may be largely initiated by inflammatory and pro-oxidant events leading to protein misfolding, demyelination, and mitochondrial dysfunction. The wide range of consequences of arsenic exposure is described in detail. Possible transgenerational epigenetic effects are also considered and discussed.

The critical review by Harry [4] examines the relationships between neurons and microglia. The beneficial aspects of these are vital for the maintenance of effective neuron function. However, the excessive response of glia to some exogenous factors may lead to non-productive inflammatory changes and the production of excess reactive oxygen species. The molecular basis underlying the initiation of such sterile immune responses is outlined. The beneficial and harmful consequences of the phagocytic propensity of microglia are analyzed in detail. The relation of these characteristics to several neurodegenerative diseases, including stroke, is discussed. The article concludes with a description of age-related loss of efficient targeting by microglia. Glial deficits incurred during senescence may form a platform facilitating the onset of specific neurodegenerative disorders. Their prior experience may lead to an inability to “forget” prior experiences, leading to an accumulation of non-selective inflammatory changes.

The article by Tinkov et al. [5] summarizes recent work on manganese neurotoxicity. There may be several primary sites of action for this metal, leading to a cascade of secondary effects on many intracellular targets. Multiple metabolic pathways respond to manganese. The interpretation of manganese’s effects on these is complicated as, in addition to being toxic at higher levels, manganese is also an essential element. Induction of oxidative stress and non-selective inflammatory events are certainly components of manganese toxicity, along with the reduction of neurogenesis and induction of apoptosis. The emphasis of manganese toxicity on the dopaminergic system remains unresolved, but there is evidence that the transcription of tyrosine hydroxylase and some dopamine receptors may be especially vulnerable sites.

Some general themes emerge from these reports. These include the primacy of excessive and non-target inflammatory and oxidative events, together with the commonality of mitochondrial dysfunction and the promotion of apoptotic processes. All of these changes also characterize normal aging, and it is likely that the consequences of neurotoxic exposures and neurosenescence are mutually reinforcing. What is often lacking is an understanding of the sequence of adverse changes and the distinction between primary and secondary responses.

Several of the articles also discuss the possibility of remedying deficits. Consideration of means of improving damage attributable to neurotoxic exposures should ultimately be a key component of such research.
